# Prasinezumab: A Bayesian Perspective on Its Efficacy

**DOI:** 10.1002/mds.30129

**Published:** 2025-01-27

**Authors:** Mirella Russo, Tommaso Costa, Dario Calisi, Stefano L. Sensi

**Affiliations:** ^1^ Department of Neuroscience, Imaging, and Clinical Sciences “G. d'Annunzio” University of Chieti‐Pescara Chieti Italy; ^2^ Institute of Neurology “SS. Annunziata Hospital,” ASL Lanciano‐Vasto‐Chieti Chieti Italy; ^3^ Center for Advanced Studies and Technology “G. d'Annunzio” University of Chieti‐Pescara Chieti Italy; ^4^ GCS‐fMRI, Koelliker Hospital and Department of Psychology University of Turin Turin Italy; ^5^ Institute for Advanced Biomedical Technologies “G. d'Annunzio” University of Chieti‐Pescara Chieti Italy

**Keywords:** prasinezumab, Parkinson's disease, Bayesian, disease‐modifying therapy, synuclein

## Introduction

### α‐Synuclein: Gain of Function Versus Loss of Function

**FIG. 1:**
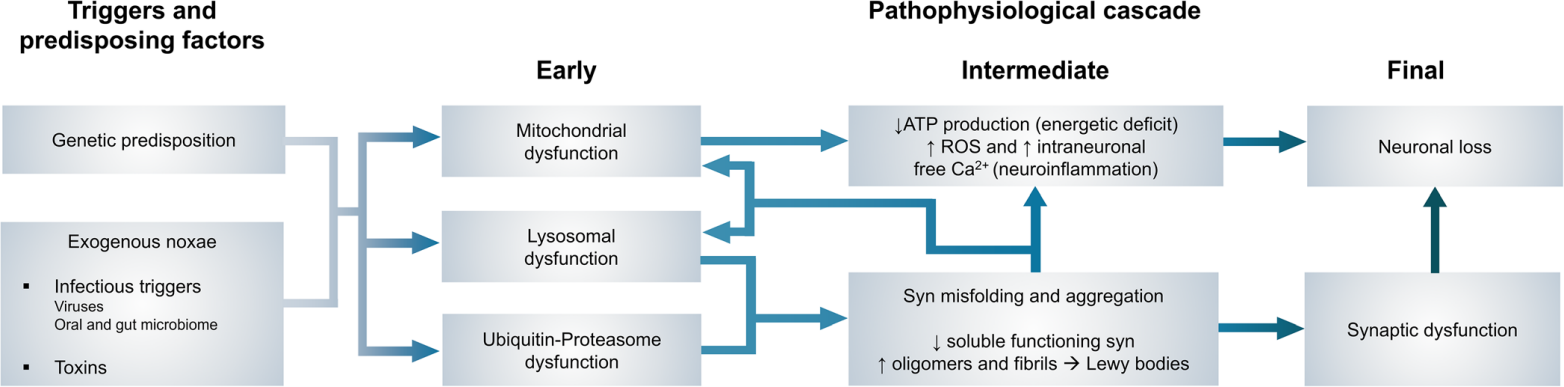
Parkinson's disease (PD) pathophysiological cascade. This figure illustrates the stepwise progression of the pathophysiological cascade ultimately leading to synaptic dysfunction and neural loss in PD. The left section highlights the early triggers and predisposing factors, including genetic predispositions and exogenous *noxae*, such as infectious triggers (eg, viruses, oral and gut microbiome) and toxins. These factors initiate early cellular dysfunctions, including mitochondrial, lysosomal, and ubiquitin‐proteasome system impairments. As the cascade progresses (center section), these dysfunctions converge on intermediate mechanisms such as reduced ATP production (energetic deficit), increased ROS and intraneuronal free Ca^2+^ levels (neuroinflammation), and α‐synuclein (α‐syn) misfolding and aggregation. The latter leads to a loss of functional soluble α‐syn (loss of function), the formation of toxic (gain‐of‐function) oligomers and fibrils, and ultimately the development of Lewy bodies. The right section depicts the final stages of the cascade, characterized by synaptic dysfunction and neuronal loss, which underlie clinical disease manifestations. As shown above, the α‐syn misfolding and aggregation in Lewy bodies is just one step of many. The weight of the loss of physiological function in synaptic homeostasis should not be neglected compared with the pathogenic role played by protein aggregates. Furthermore, neurodegeneration in PD can follow paths independent of α‐syn–driven proteinopathy. These paths must be acknowledged and considered valuable therapeutic targets. ATP, adenosine triphosphate; Ca^2+^, intraneuronal free calcium ions; ROS, reactive oxygen species. [Color figure can be viewed at wileyonlinelibrary.com]

Parkinson's disease (PD) is a progressive neurodegenerative disorder featuring aberrant aggregation of α‐synuclein (a presynaptic protein implicated in physiological synaptic vesicle trafficking and neurotransmitter release) as a critical modulator of the disease.^1^, ^2^ This notion has been further substantiated by the discovery of autosomal dominant variants of PD associated with mutation or triplication of the α‐synuclein gene *SNCA* (PARK1 and PARK4, respectively).^3^ In PD, α‐synuclein undergoes conformational changes (Fig. 1), leading to the formation of insoluble fibrils and intracellular inclusions known as Lewy bodies.^4^ One of the leading pathogenic theories postulates that these aggregates propagate in a prion‐like manner, spreading throughout the brain in a pattern that correlates with disease progression.^5^, ^6^, ^7^, ^8^ Although the link between α‐synuclein anomalies and PD has been established, the modalities of this association are still unclear. A revised approach is considering that both a loss of function of a physiological (monomeric) protein^9^ and a gain of abnormal function could play a role.^10^, ^11^ On the one hand, α‐synuclein oligomers and fibrils exhibit toxic properties as these aggregates disrupt cellular homeostasis, impair mitochondrial function, and induce oxidative stress, neuroinflammation, and synaptic dysfunction, ultimately leading to neuronal dysfunction and death.^10^ On the other hand, it has also been hypothesized that the loss of function of α‐synuclein (*synucleinopenia*)^9^ may consistently affect PD pathophysiology. Due to its main localization at presynaptic terminals, the protein regulates neurotransmitter release and synaptic vesicle dynamics. Thus, its loss of function may impair synaptic transmission. Because α‐synuclein is predominantly expressed in dopaminergic neurons, its loss of function may dysregulate dopaminergic signaling pathways.^12^ Furthermore, α‐synuclein is involved in protein degradation pathways, including the ubiquitin‐proteasome system and autophagy‐lysosomal pathway.^13^ According to the model, a defective α‐synuclein could favor the accumulation of misfolded proteins and protein aggregates, characteristic of PD pathology, thereby generating mixed neuropathology.^14^ α‐Synuclein also interacts with mitochondria, influencing mitochondrial dynamics and function. Thus, a loss of function may disrupt mitochondrial bioenergetics and quality‐control mechanisms, leading to mitochondrial impairment and oxidative stress.^15^ Moreover, α‐synuclein has been linked to neuroprotection against excitotoxicity.^16^ Therefore, its loss of function could compromise neuronal resilience to stressors, making dopaminergic neurons more susceptible to degeneration.

Several technical issues challenge the toxic role of α‐synuclein. For instance, overexpression of α‐synuclein due to *SNCA* gene multiplication does not necessarily lead to toxicity. High levels of α‐synuclein are associated with better outcomes in some studies.^9^ Preclinical studies in knocked‐out α‐synuclein mice produced no substantial adverse effects. α‐Synuclein knocked‐out mice were fertile and showed normal neurodevelopment. However, dopaminergic dysfunction and abnormal motor responses were observed.^17^ The prion‐like propagation of α‐synuclein spreads is also questioned. It should also be underlined that the formation of α‐synuclein aggregates is a passive, not active, process controlled by thermodynamic principles. Oligomers are indicated as toxic agents, but their real role is elusive because most experimental settings use supersaturated protein levels in which the presence of oligomers is transient, and they usually revert to monomers rather than forming fibers. This challenges the idea that oligomers can persist long enough to exert toxicity.^9^ This evidence has challenged the gain‐of‐function hypothesis and suggested that α‐synuclein aggregation into Lewy bodies may be a protective mechanism rather than a toxic process.

The recent development of disease‐modifying therapies (DMTs) for PD has been a turning point. DMTs targeting α‐synuclein are also crucial to confirm the “proteinopathy hypothesis,” whereas negative results could successfully be the Popperian “falsification element” against it (or at least could demonstrate that the matter is far more complex than a mere protein loss of function; see Fig. 1). One complicating factor is that currently, there are no precise biomarkers for PD, and the assessment of therapy‐related changes can be challenging to assess in the short time span of clinical trials. Currently, no DMT has been approved for clinical use.

More importantly, PD is still regarded as a disease, even though it better fits the construct of a syndrome.^18^ That point is worthwhile because it implies the challenging realization of homogeneous cohorts in PD clinical trials.

### Prasinezumab, the Long‐Sought First DMT?

Prasinezumab (PRX002) is a monoclonal immunoglobulin G1 antibody designed to selectively target the soluble and insoluble aggregated forms of α‐synuclein, such as oligomers and fibrils, while sparing the physiological, soluble forms of the protein^19^ (see Supporting Information Data S1 [Preclinical Studies on Prasinezumab section] for the results of preclinical studies).

In 2022, the PASADENA phase 2 trial^20^ assessed the efficacy of prasinezumab in patients with early‐stage PD. The results were disappointing.^20^ Participants were randomly assigned to receive either a placebo or the compound (1500 or 4500 mg) every 4 weeks for 52 weeks. The primary outcome measured changes in the Movement Disorder Society–revised Unified Parkinson's Disease Rating Scale (MDS‐UPDRS) scores, with secondary outcomes assessing dopamine transporter levels via single‐photon emission computed tomography. Among 316 participants, the MDS‐UPDRS scores and dopamine transporter levels did not differ when comparing the treatment and placebo groups.^20^ Overall, prasinezumab did not significantly affect PD progression compared with placebo, and optimism faded. Nonetheless, a new post hoc analysis was carried out and recently published.^21^ The post hoc analysis of the PASADENA study showed that the compound might slow motor progression in predefined subpopulations of patients with early‐stage PD.^21^ Although the original study did not meet its primary endpoint (changes in MDS‐UPDRS Parts I + II + III scores),^20^ in the subset of rapidly progressing patients,^21^ compared with placebo, drug‐treated participants showed less worsening in motor signs (MDS‐UPDRS Part III). The ongoing PADOVA study (NCT04777331) further investigates the impact of prasinezumab on motor progression in early‐stage PD. However, methodological limitations must be accounted for, and longer trials might be required to observe effects in slowly progressing populations.

Nonetheless, the recent post hoc analysis of the PASADENA trial raised many questions about the drug's efficacy because it indicated a numerical effect on motor progression in specific subpopulations (ie, fast progressing phenotypes). However, the lack of significant overall effects raises concerns about the robustness of these findings. First, the distinction between “diffuse malignant” and “nondiffuse malignant” phenotypes was made retrospectively and was data driven, which may have introduced a retrospective selection bias.^21^ The identification of “diffuse malignant” phenotypes at baseline with those with “more rapid” worsening is also problematic, because it conflates baseline features with outcomes. Moreover, the assessment of rapid progression was limited to the time of the observation and could carry a time‐window bias and, as acknowledged by the authors of the original study,^21^ could reflect a different sensitivity to “signal‐to‐noise ratio” of the clinician's assessment (ie, the greater the change at scales, the greater the amplification of its perception, and vice versa). Furthermore, the prescription monoamine oxidase B inhibitors at baseline is not necessarily associated with a rapid progression of the disease but could depend on different factors, including the prescriber's preferences. Lastly, the mechanism of action of prasinezumab should counter a pathophysiological event that occurs relatively early in the disease course.^19^ Therefore, the association between drug efficacy and rapidity of clinical worsening, which is not necessarily associated with higher degree of α‐synuclein deposition, is somehow confusing.

Therefore, we used a Bayesian approach to test the effectiveness of prasinezumab for PD treatment. This provides a new angle on interpreting the trial results and offers critical elements for a constructive debate.

## Materials and Methods

### Bayes Factor

Data analysis was performed using a Bayesian framework, which allows us to quantify the evidence for competing hypotheses based on the observed data. Specifically, we employed the Bayes factor (BF), a measure that compares how well two hypotheses, such as the null hypothesis ($H_{0}$) and the alternative hypothesis ($H_{1}$), predict the observed data. In its simplest form, the BF can be thought of as a ratio of likelihoods, sometimes called the likelihood ratio. The hypothesis that better explains the observed data is said to have more evidence in its favor. The formula for the BF is as follows:

$$BF_{01}=\frac{L\left(\text{data}|H_{0}\right)}{L\left(\text{data}|H_{1}\right)}$$

where L represents the likelihood or how probable the data are under each hypothesis. This ratio quantifies how much the data support the null hypothesis ($H_{0}$) over the alternative hypothesis ($H_{1}$).

In our case, we used summary statistics to calculate the BF, particularly focusing on *t* statistics. Given the sample size (N), adjusted mean difference, and standard error (SE) in each condition, we derived the corresponding *t* values. Using these *t* values, we calculated the BF based on both the null hypothesis (no effect) and the alternative hypothesis (there is an effect). The equation used for this calculation is

$$B_{01}=\begin{array}{l} {\left(1+\frac{t^{2}}{\nu }\right)}^{-\frac{\left(\nu +1\right)}{2}}/\int_{0}^{\infty }{\left(1+N_{0}g\right)}^{-\frac{1}{2}}\\ (1{+\;\frac{t^{2}}{\left(1+N_{0}g\right)\nu })}^{-\frac{\nu +1}{2}}{\left(2\pi \right)}^{-\frac{1}{2}}g^{-\frac{3}{2}}e^{-\frac{1}{2g}}\mathrm{dg} \end{array}$$

where

- ν=n1+n2−2 is the degrees of freedom, and
- $N_{0}=\frac{n1n2}{n1+n2}$ is the effective sample size.

This equation helps to calculate the BF for independent groups *t* test based on the most frequent *t* statistic. The null hypothesis ($H_{0}$) in this test assumes that the population means of two independent groups are equal. Several assumptions underpin this method: the observations in both groups should be random samples, the dependent variable is normally distributed within each population, and the population variances should be equal (ie, the spreads of values in both groups are similar).

The BF ($BF_{01}$) can take values between 0 and infinity. When $BF_{01}<1$, the evidence favors the alternative hypothesis ($H_{1}$); when $BF_{01}>1$, the evidence supports the null hypothesis ($H_{0}$). Importantly, this approach allows for a full comparison of both hypotheses, modeling “what the data should look like when there is an effect.”

The strength of the evidence depends on how far from 1 the BF is. Jeffreys (1939) proposed conventional thresholds: a BF greater than 3 or less than 1/3 is considered substantial evidence favoring one hypothesis over the other. Anything between 1/3 and 3 is interpreted as weak or anecdotal evidence. For example, if $BF_{01}=6$, this suggests that the null hypothesis ($H_{0}$) is six times more likely than the alternative hypothesis ($H_{1}$). In terms of posterior probabilities, this translates to an 86% probability for the null hypothesis ($H_{0}$) (calculated as $P_{01}=\frac{BF_{01}}{BF_{01}+1}=\frac{6}{7}$), leaving a 14% probability for the alternative hypothesis ($H_{1}$).

All Bayesian analyses were performed using the JASP software.^22^ All results were also tested for robustness using a BF robustness check (see Supporting Information Data S1, Methods: Bayes Factor section).

## Results

This study employed a Bayesian approach to examine the impact of prasinezumab on the progression of PD symptoms and signs. We used the BF in hypothesis testing. The BF is inherently comparative: it weighs the support for one model against that of another. Moreover, BFs do so by fully conditioning on the observed data. Otherwise, the *P* value depends on hypothetical outcomes that are more extreme than those observed in the sample. Such practice violates the likelihood principle and results in inconsistent or paradoxical conclusions. The BF can quantify evidence in favor of the null hypothesis. In the Bayesian framework, no special status is attached to either of the hypotheses under test; the BF assesses each model's predictive performance and expresses a preference for the model that made the most accurate forecasts. The fact that the BF can quantify evidence in favor of the null hypothesis can be of substantive importance. For instance, the hypothesis of interest may predict the absence of an effect across a varying set of conditions. Quantifying the null hypothesis is also important to learn whether the observed data provide evidence of absence or absence.

Specifically, the possible outcomes of the BF can be assigned to three discrete categories: (1) evidence in favor of $H_{1}$ (ie, evidence in favor of the presence of an effect), (2) evidence in favor of $H_{0}$ (ie, evidence in favor of the absence of an effect), and (3) evidence that favors neither $H_{1}$ nor $H_{0}$. Instead, the *P* value cannot provide a measure of evidence in favor of the null hypothesis. Finally, the BF is not affected by the sampling plan, that is, the intention with which the data were collected. This irrelevance follows from the likelihood principle, and it means that BFs may be computed and interpreted even when the intention with which the data are collected is ambiguous, unknown, or absent. All these advantages are not available if a classical analysis is performed as was done for the PASADENA trial data.

Based on the findings shown in the first table of the source article^21^ (Table 1), a Bayesian analysis of the results obtained in these subpopulations was carried out. The results of the Bayesian analysis are shown in Table 2. The posterior probability column on the drug's effectiveness indicates no major and clinically relevant difference between the placebo and treated groups. The lack of efficacy applies to the population with and without rapid disease progression. The probability of efficacy is consistently less than 50% and near 50% only for the data‐driven subphenotype diffuse malignant subgroup. The results support the notion that the drug's effects cannot be safely assessed.

**TABLE 1:** Table used for the Bayesian analysis, adapted from Figure 1 of the original paper by Pagano et al., demonstrating the impact of prasinezumab on motor progression, as assessed by the MDS‐UPDRS Part III (hypothetical strategy), within the main predefined sub‐populations

Category	Subgroup	Total n	Adjusted mean difference	80% CI
MAO‐B inhibitor	Yes	115	−2.66	(−4.87, −0.45)
	No	201	−0.87	(−2.69, 0.94)
Hoehn and Yahr stage	2	238	−2.55	(−4.19, −0.9)
	1	78	3.14	(0.32, 5.95)
RBDSQ	**≥**5	85	−2.76	(−5.78, 0.25)
	<5	230	−1.03	(−2.63, 0.57)
Data‐driven subphenotype	Diffuse malignant	59	−7.86	(−12.9, −2.82)
	Nondiffuse malignant	257	−0.77	(−2.2, 0.66)

The data from Pagano et al^21^ were used for the Bayesian analysis. Participants with and without MAO‐B inhibitors at baseline, participants with Hoehn and Yahr stages 1 and 2, participants with and without RBDSQ, and participants with diffuse and nondiffuse malignant phenotypes (data‐driven subphenotype) were included.

Abbreviations: CI, confidence interval; MAO‐B, monoamine oxidase B; RBDSQ, Rapid Eye Movement Sleep Behavior Disorder screening questionnaire.

**TABLE 2:** Results of the Bayesian data analysis

Category	Subgroup	$BF_{10}$	$P_{10}$ (%)
MAO‐B inhibitor	Yes	0.44	30.75
	No	0.13	11.66
Hoehn and Yahr stage	2	0.69	40.90
	1	0.44	30.65
RBDSQ	**≥**5	0.33	24.59
	<5	0.14	12.51
Data‐driven subphenotype	Diffuse malignant	1.17	53.92
	Nondiffuse malignant	0.13	11.11

The $BF_{10}$ is the Bayes factor (BF) of the evidence that $H_{1}$ respects $H_{0}$, the null hypothesis; $P_{10}$ is the posterior evidence that the $H_{1}$ hypothesis respects the null hypothesis.

Abbreviations: MAO‐B, monoamine oxidase B; RBDSQ, Rapid Eye Movement Sleep Behavior Disorder screening questionnaire.

**FIG. 2:**
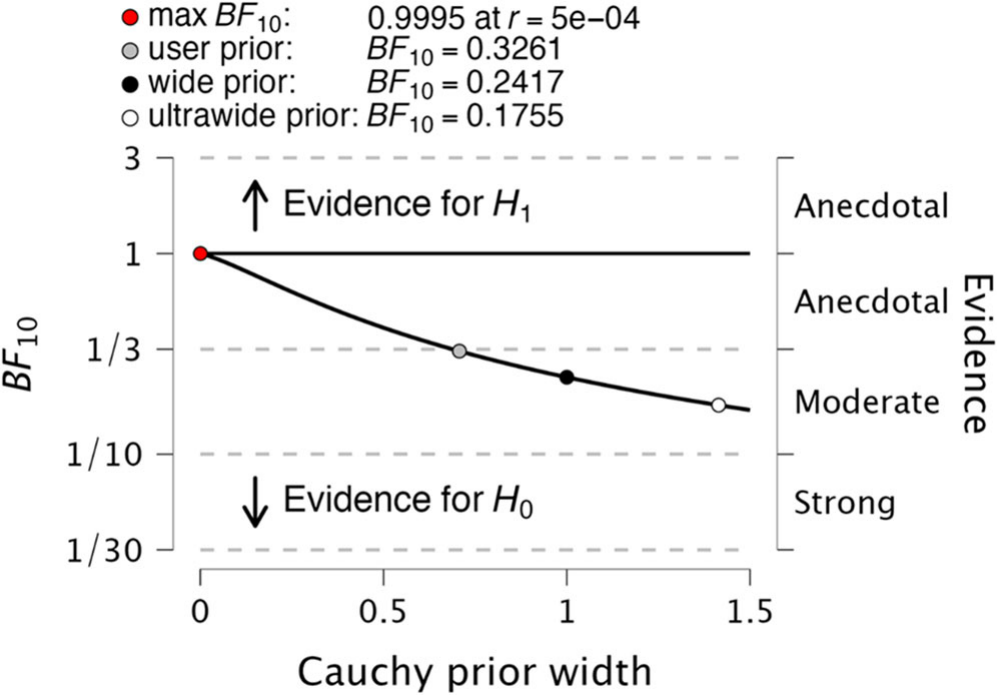
Robustness. The graph illustrates JASP's output. The graph shows the value of $BF_{10}$ as a function of the width (ie, scale) of the Cauchy prior. Only one graph is shown for a particular group as an example. For the wide prior, γ = 1, and for the ultrawide prior, γ = √2, the user prior corresponds to the scale chosen in the current analysis, which is 0.707. The arrows indicate ranges in which the $BF_{10}$ means there is evidence for $H_{1}$ (ie, values > 1) and in which the $BF_{10}$ indicates there is evidence for $H_{0}$ (ie, values < 1). In general, the gradients are the same. As shown, there is no variation between the two hypotheses as the a priori varies but only an increase in the Bayes factor (BF) on the same hypothesis. See Supporting Information Data S1 (Preclinical Studies on Prasinezumab section) for additional analyses. [Color figure can be viewed at wileyonlinelibrary.com]

The findings are robust, as depicted in Figure 2, which shows no variation between the two hypotheses as the a priori changes; instead, there is only an increase in the BF for the same hypothesis. Additional analyses are described in Supporting Information Data S1 (Additional Bayesian Analyses section) and support the main results. Thus, there is no substantial evidence of a difference in efficacy between subpopulations with or without rapid progression. Ultimately, these results are consistent with the phase two PASADENA trial, which did not reach the primary end point at 52 weeks. In conclusion, the exploratory analysis to assess whether prasinezumab generates greater benefits on motor progression in prespecified subgroups with faster motor progression using the BF resulted in no supporting evidence.

## Discussion

The negative findings related to the anti–α‐synuclein approach confirm the many still unknown physiological and pathophysiological mechanisms controlled by the protein.

Incorporation of the Bayesian approach in analyzing the efficacy of prasinezumab for the treatment of PD offers a number of important strategies for ongoing and future clinical trials, like adaptive designs to allow real‐time decision‐making and optimizing the trial parameters.^23^ The Bayesian framework also provides a probabilistic approach that quantifies uncertainty in treatment effects, thereby better informing decisions by stakeholders about modifications in trials, such as early stopping and expansion or changes in allocation ratios.^24^ Furthermore, Bayesian hierarchical models enable the assessment of individual variability in treatment responses, providing the groundwork for personalized treatment strategies.^25^

Beyond statistical and methodological issues, an additional point that should be considered when discussing the unsuccessful attempts to reach DMTs pertains to the disconnect between therapeutic strategies that are envisioned and tested in “clean and sanitized” clinical trials and the more difficult challenges offered by real‐world settings. As recently discussed by Brett K. Beaulieu‐Jones et al,^26^ research populations are studied among actively recruited individuals who often receive earlier diagnoses and comply with more consistent follow‐ups. In contrast, in real‐world populations, patients are diagnosed later in life and often exhibit a more rapid progression because of a combination of selection bias, multiple‐hit comorbidity, late access to care, and intrinsic population differences.^26^ The study also highlighted bias in data collection. The mode of data gathering (actively recruited vs. passively recorded) introduces biases that must be carefully considered in clinical trial design and real‐world analyses, ultimately affecting the validity of clinical outcomes.

Somehow, the PASADENA trial mirrors findings from the Alzheimer's field in which multiple clinical trials targeting a single protein, amyloid, generated modest or null effects, substantially failing as disease‐modifying intervention.^27^ In that regard, novel insights are promoting a reconceptualization of PD itself as more than a mere “synucleinopathy”, but rather a heterogeneous disorder arising from the convergence of multiple pathological processes but ultimately giving a variety of clinical manifestations. Most likely, “there is more than one Parkinson's Disease,”^28^ and our collective efforts should focus on dissecting the convergent and divergent mechanisms that act inside or outside the central nervous system.

## Author Roles

Research project: A. Conception, B. Organization, C. Execution; Statistical analysis: A. Design, B. Execution, C. Review and critique; Manuscript: A. Writing of the draft, B. Review and critique.

MR: 1A, 1B, 1C, 2C, 3A, 3B.

TC: 1A, 1C, 2A, 2B, 2C, 3A, 3B.

DC: 1A, 1C, 2C, 3A, 3B.

SLS: 1A, 1B, 1C, 2C, 3A, 3B.

## Full Financial Disclosure for the previous 12 months

S.L.S. is supported by research funding from the Italian Department of Health (RF‐2013–02358785 and NET‐2011‐02346784‐1), from the AIRAlzh Onlus (ANCC‐COOP), from the Alzheimer’s Association—Part the Cloud: Translational Research Funding for Alzheimer’s Disease (18PTC‐19‐602325) and the Alzheimer’s Association—GAAIN Exploration to Evaluate Novel Alzheimer’s Queries (GEENA‐Q‐19‐596282).

## Supporting information


**Data S1.** Supporting Information.

## Data Availability

The data that support the findings of this study are available from the corresponding author upon reasonable request.
