# Supplementary material for: Prasinezumab: A Bayesian Perspective on Its Efficacy
Source: Mov Disord. 2025 Jan 27;40(4):619–24. doi: 10.1002/mds.30129 (PMC12006898; doi:10.1002/mds.30129)

**S1 - Preclinical Studies on prasinezumab**

Derived from the murine monoclonal antibody 9E4, PRX002 was developed based on immunization experiments demonstrating the efficacy of antibodies targeting carboxyl terminus epitopes of α‐synuclein in reducing neuronal accumulation and behavioral deterioration in animal models (Schenk et al., 2017). In the first phase 1 trial (randomized, double‐blind, placebo‐controlled, carried out in 40 healthy volunteers), single intravenous doses of PRX002 were safe and well-tolerated up to the highest tested dose (30 mg/kg), with no serious adverse events reported. PRX002 significantly reduced free serum α‐synuclein levels within one hour post-infusion, persisting for up to four weeks after a single dose. No immunogenicity (anti‐PRX002 antibodies) was observed. However, no analyses on CSF were carried out in this first human trial (Schenk et al., 2017). Subsequently, a phase 1b multicenter, randomized, double-blind, placebo-controlled trial involved sequential enrollment into escalating-dose cohorts (up to 60 mg/kg) receiving intravenous infusions of PRX002 or placebo every 28 days (Jankovic et al., 2018). Safety assessments only revealed mild adverse events (AEs). Rapid, dose-dependent reductions in free serum α-synuclein levels were confirmed in post-infusion sampling (Jankovic et al., 2018). Although reductions in free serum α-synuclein levels were rapid, dose-dependent, and sustained following administration of PRX002, some limitations should be considered. The absence of validated assays for directly assessing aggregated α-synuclein levels in cerebrospinal fluid (CSF) and imaging methods to monitor intracellular α-synuclein pathology in PD patients' brains impair the evaluation of the drug's effect on the central nervous system (CNS). PRX002's concentration in the CSF may not be sufficiently high to effectively engage monomeric α-synuclein species, potentially explaining the lack of observed impact on free α-synuclein levels in CSF and the technical limitations (Jankovic et al., 2018).

**References**

Jankovic, J., Goodman, I., Safirstein, B., Marmon, T. K., Schenk, D. B., Koller, M., Zago, W., Ness, D. K., Griffith, S. G., Grundman, M., Soto, J., Ostrowitzki, S., Boess, F. G., Martin-Facklam, M., Quinn, J. F., Isaacson, S. H., Omidvar, O., Ellenbogen, A., & Kinney, G. G. (2018). Safety and Tolerability of Multiple Ascending Doses of PRX002/RG7935, an Anti–α-Synuclein Monoclonal Antibody, in Patients With Parkinson Disease. *JAMA Neurology*, *75*(10), 1206. https://doi.org/10.1001/jamaneurol.2018.1487

Schenk, D. B., Koller, M., Ness, D. K., Griffith, S. G., Grundman, M., Zago, W., Soto, J., Atiee, G., Ostrowitzki, S., & Kinney, G. G. (2017). First-in-human assessment of PRX002, an anti-α-synuclein monoclonal antibody, in healthy volunteers. *Movement Disorders*, *32*(2), 211–218. https://doi.org/10.1002/mds.26878

**S2 - Methods: Bayes Factor**

*Robustness*

All results were also tested for robustness using a Bayes Factor Robustness Check. In Bayesian statistics, this check helps us evaluate the stability of the findings and whether the results are sensitive to changes in certain assumptions, particularly the choice of prior distributions. Essentially, this check allows us to see how much the conclusions depend on the assumptions we made about the data.

The Bayes Factor Robustness Check examines how the Bayes Factor changes when we adjust the width of the Cauchy prior distribution for the effect size. A prior distribution represents what we believe about the effect size before looking at the data, and in this case, we use a Cauchy distribution. By adjusting the 'scale' or width of this prior (i.e., how spread out our prior beliefs are), we can see how this impacts the Bayes Factor.

The scale of the Cauchy prior varies between 0 and 1.5, or even up to 2 if a wider prior is chosen by the user. Narrower priors (closer to 0) reflect more certainty about the effect size being small, while wider priors (closer to 1.5 or 2) reflect greater uncertainty, allowing for larger possible effect sizes. As the prior becomes less informative (wider), it allows for a broader range of possible effect sizes, which can affect the Bayes Factor calculation.

By conducting this robustness check, we can ensure that our results are not overly dependent on the specific choice of the prior distribution, thus providing more confidence that the findings are reliable and not driven by outliers or specific assumptions.

**S3 - Additional Bayesian analyses**

**Bayesian Independent Samples T-Test MAO-B inhibitor subgroup: yes**

| Bayesian Independent Samples T-Test | | | | | | | |  |
| --- | --- | --- | --- | --- | --- | --- | --- | --- |
| t | | n₁ | | n₂ | | BF₁₀ | |  |
| -1.547 |  | 115 |  | 115 |  | 0.444 |  |  |
|  | | | | | | | |  |

**Prior and Posterior**


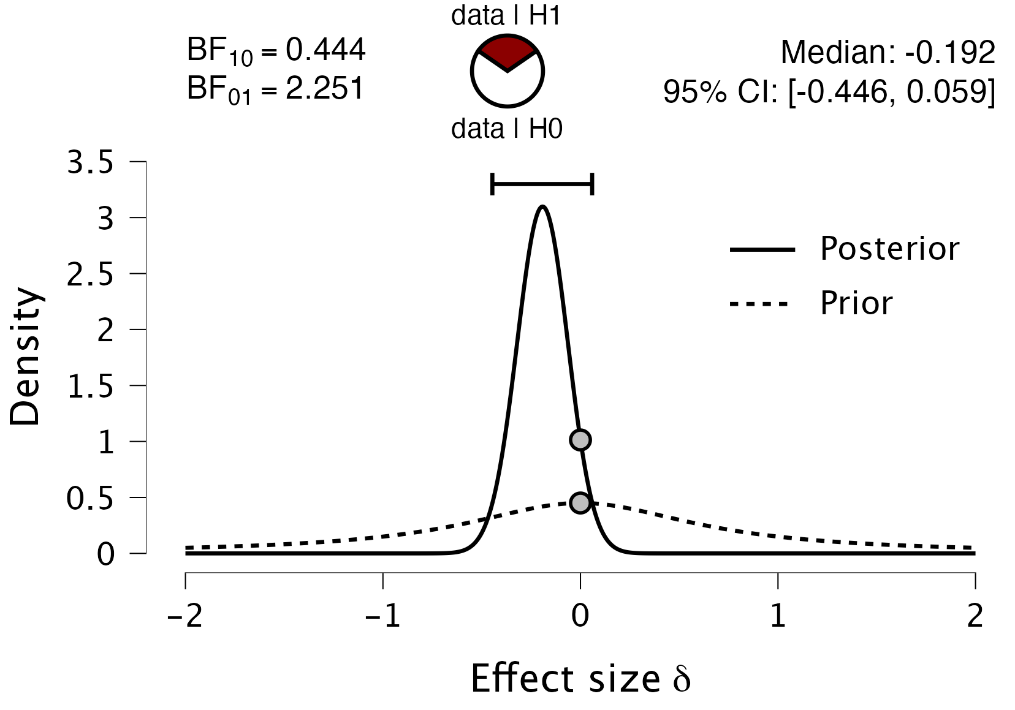


**Bayes Factor Robustness Check**


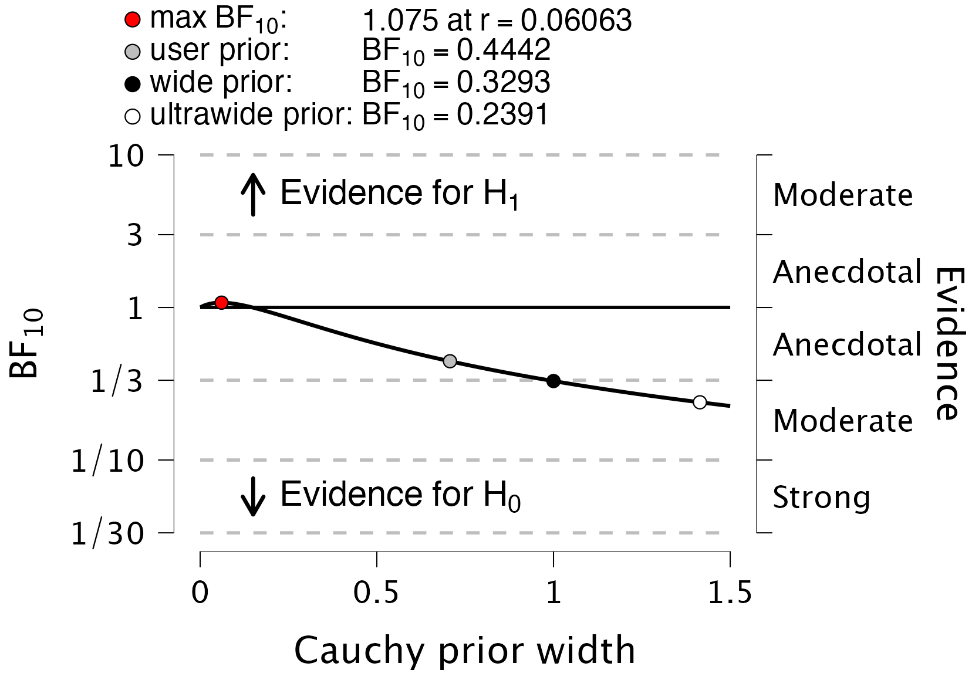


**Bayesian Independent Samples T-Test MAO-B inhibitor subgroup: no**

| Bayesian Independent Samples T-Test | | | | | | | |
| --- | --- | --- | --- | --- | --- | --- | --- |
| t | | n₁ | | n₂ | | BF₁₀ | |
| -0.610 |  | 201 |  | 201 |  | 0.132 |  |
|  | | | | | | | |

**Prior and Posterior**


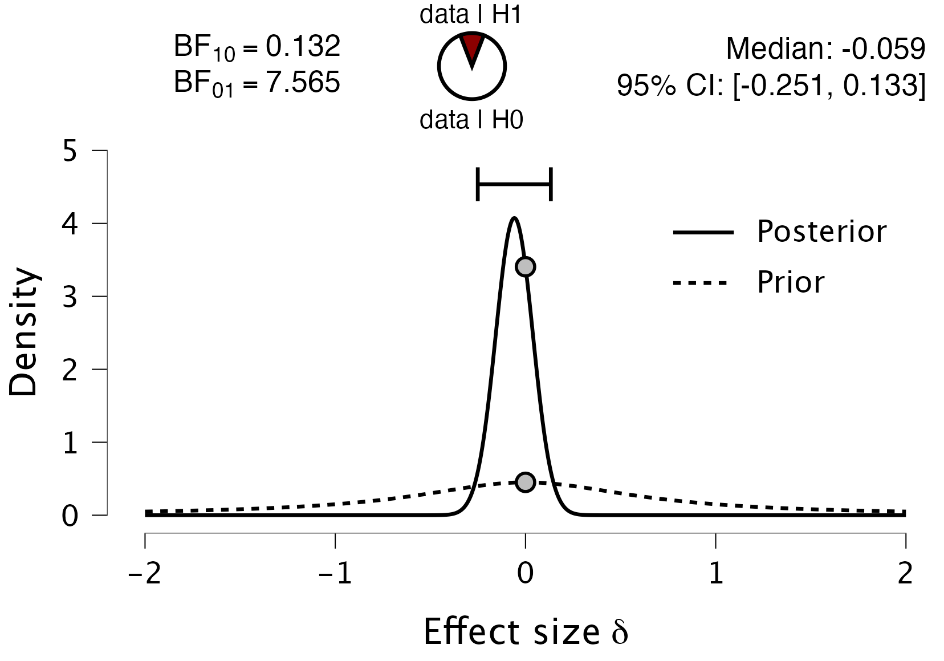


**Bayes Factor Robustness Check**


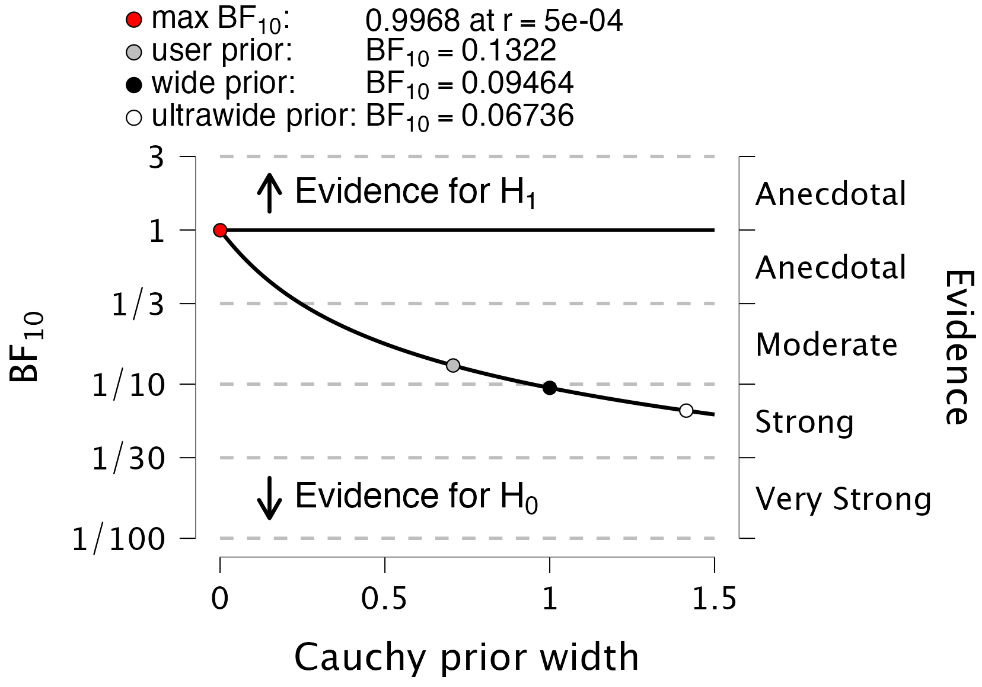


**Bayesian Independent Samples T-Test Hoehn and Yahr stage subgroup 2**

| Bayesian Independent Samples T-Test | | | | | | | |  |
| --- | --- | --- | --- | --- | --- | --- | --- | --- |
| t | | n₁ | | n₂ | | BF₁₀ | |  |
| -1.990 |  | 238 |  | 238 |  | 0.692 |  |  |
|  | | | | | | | |  |

**Prior and Posterior**


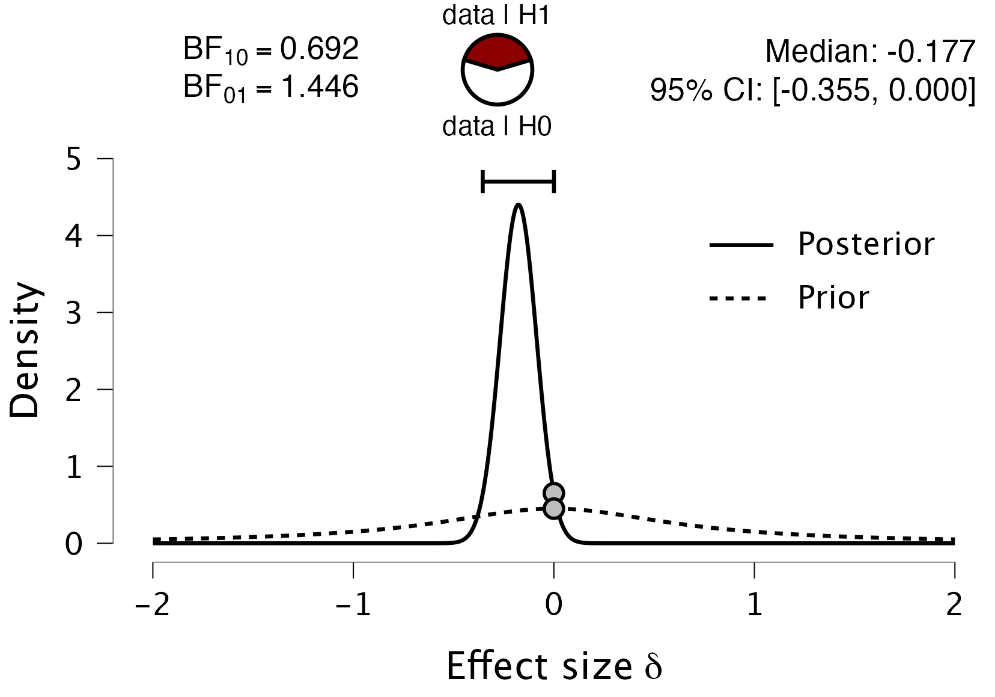


**Bayes Factor Robustness Check**


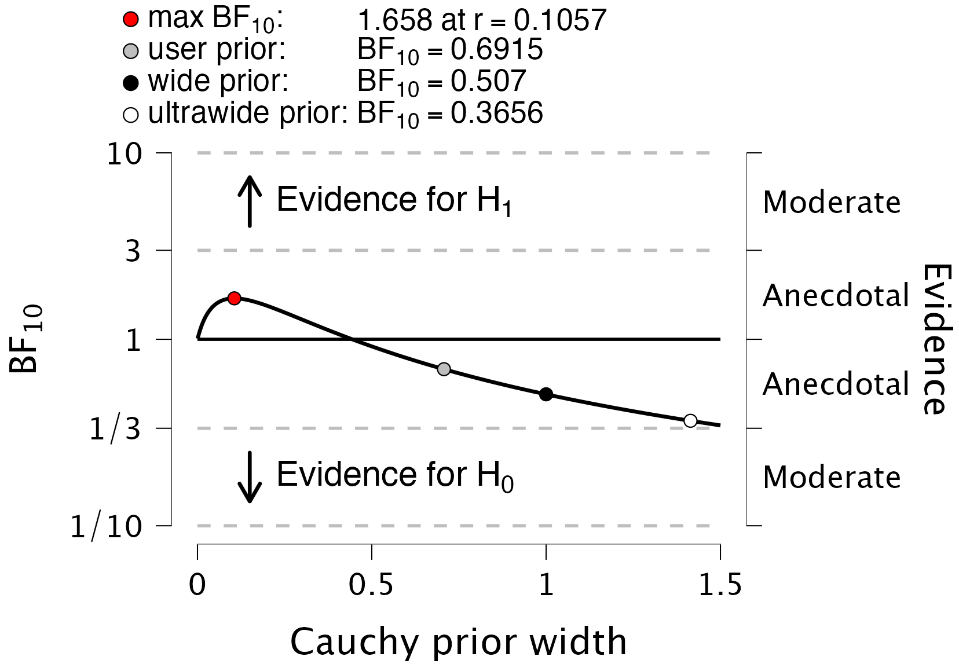


**Bayesian Independent Samples T-Test Hoehn and Yahr stage subgroup 1**

| Bayesian Independent Samples T-Test | | | | | | | | |
| --- | --- | --- | --- | --- | --- | --- | --- | --- |
| t | | n₁ | | n₂ | | BF₁₀ | |  |
| 1.430 |  | 78 |  | 78 |  | 0.442 |  |  |
|  | | | | | | | | |

**Prior and Posterior**


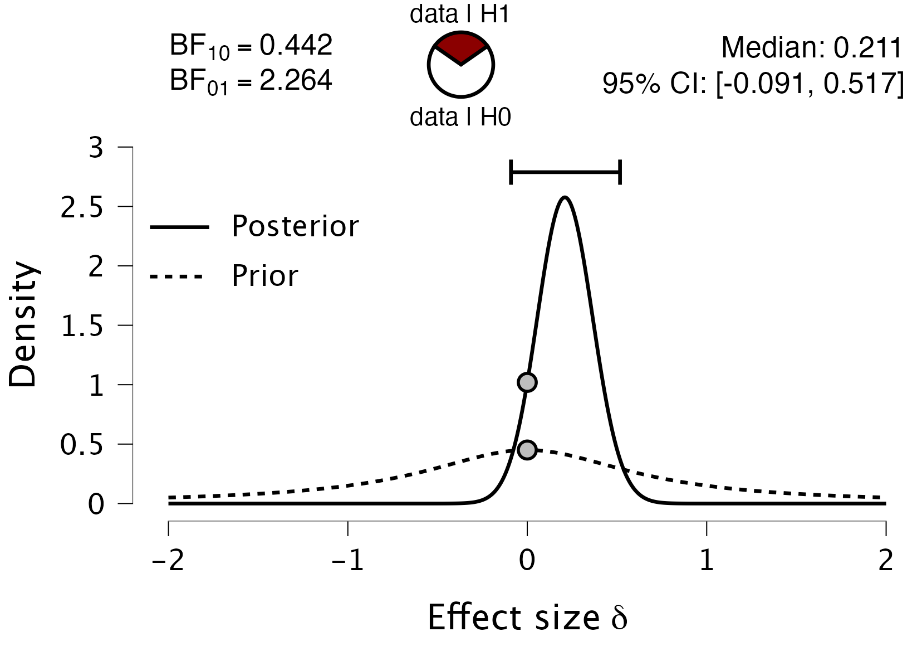


**Bayes Factor Robustness Check**


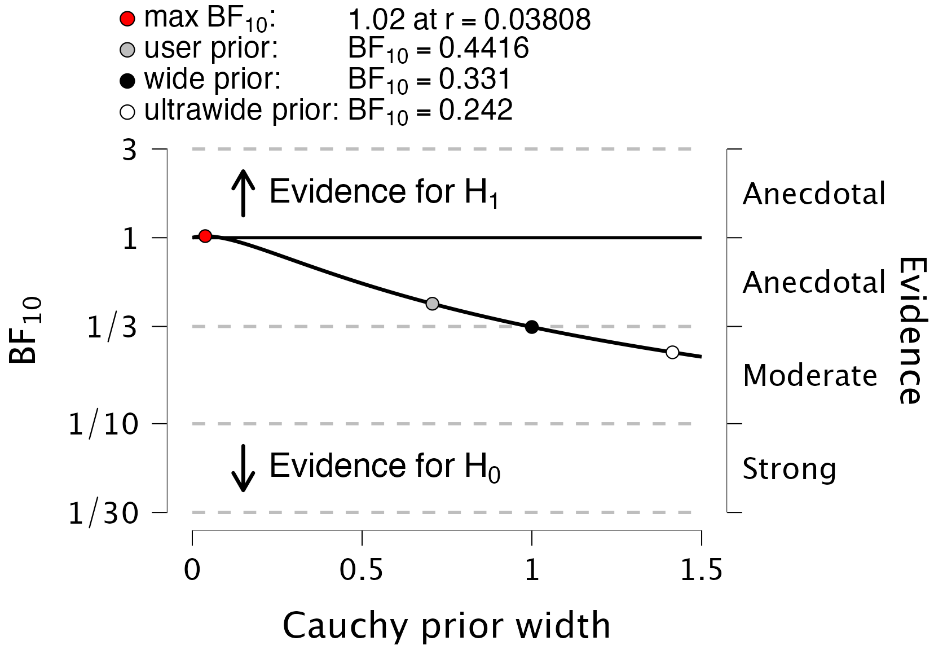


**Bayesian Independent Samples T-Test RBDSQ subgroup >=5**

| Bayesian Independent Samples T-Test | | | | | | | | |
| --- | --- | --- | --- | --- | --- | --- | --- | --- |
| t | | n₁ | | n₂ | | BF₁₀ | |  |
| -1.176 |  | 78 |  | 78 |  | 0.326 |  |  |
|  | | | | | | | | |

**Prior and Posterior**


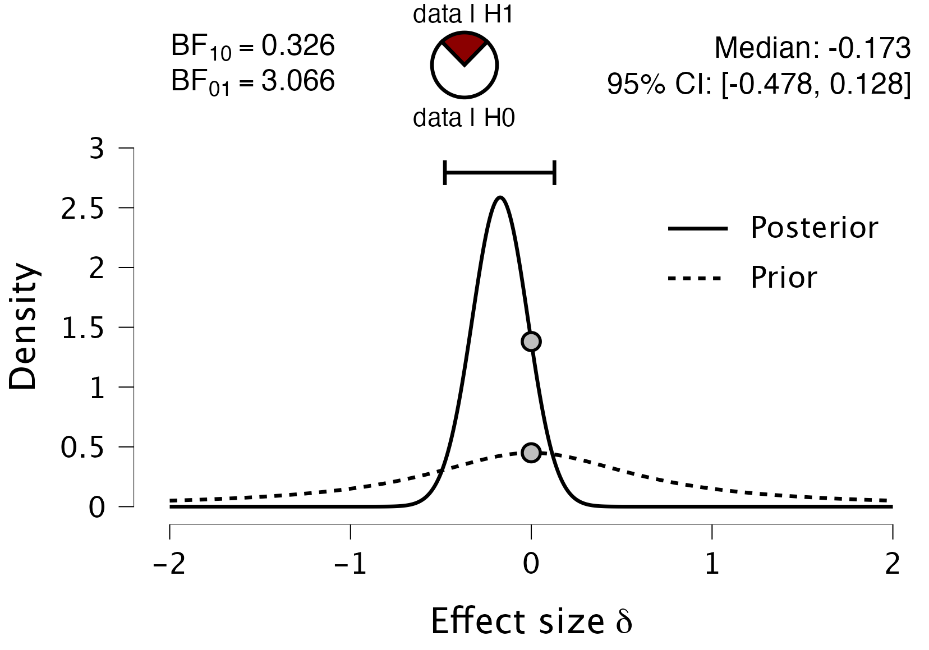


**Bayes Factor Robustness Check**


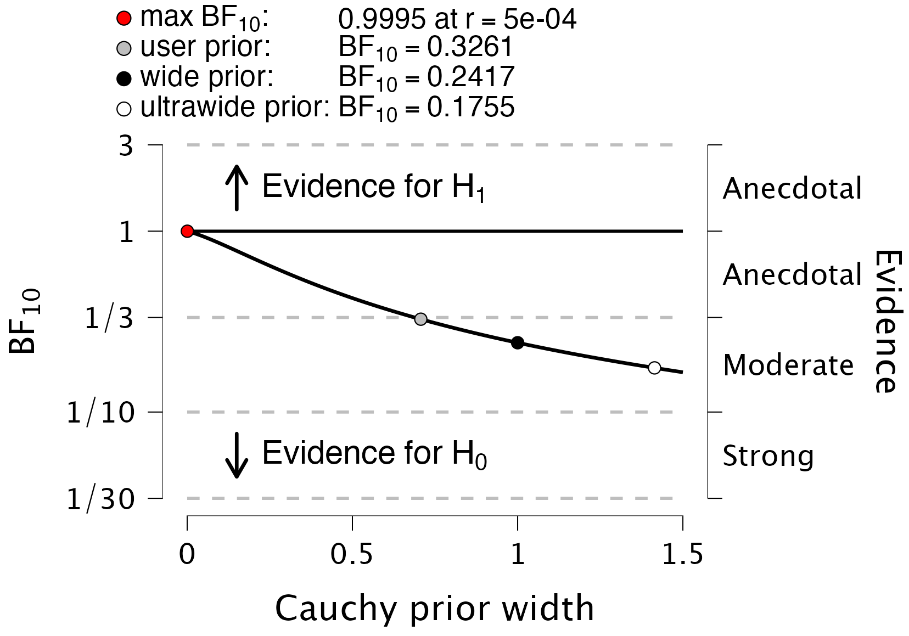


**Bayesian Independent Samples T-Test RBDSQ subgroup < 5**

| Bayesian Independent Samples T-Test | | | | | | | | |
| --- | --- | --- | --- | --- | --- | --- | --- | --- |
| t | | n₁ | | n₂ | | BF₁₀ | |  |
| -0.820 |  | 230 |  | 230 |  | 0.143 |  |  |
|  | | | | | | | | |

**Prior and Posterior**


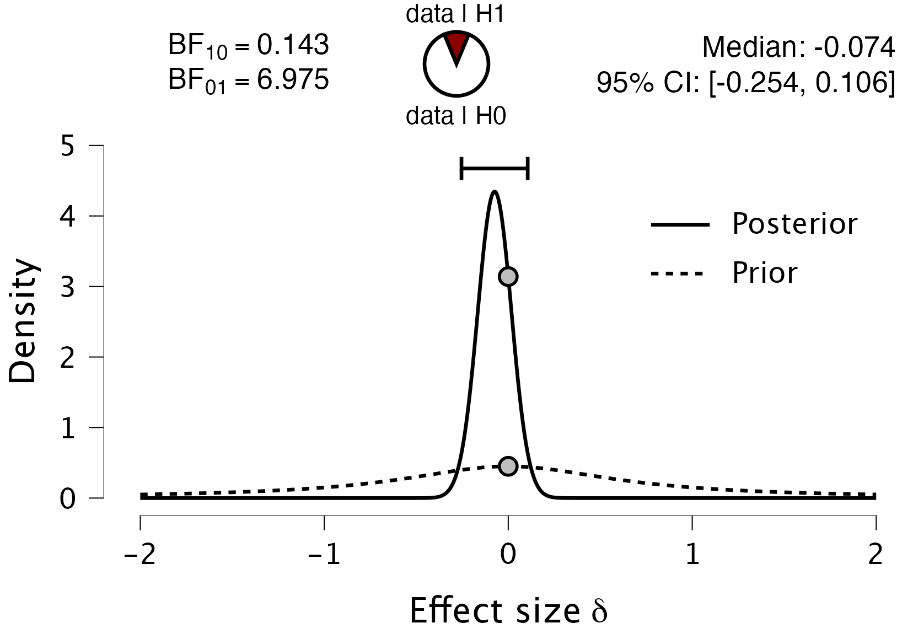


**Bayes Factor Robustness Check**


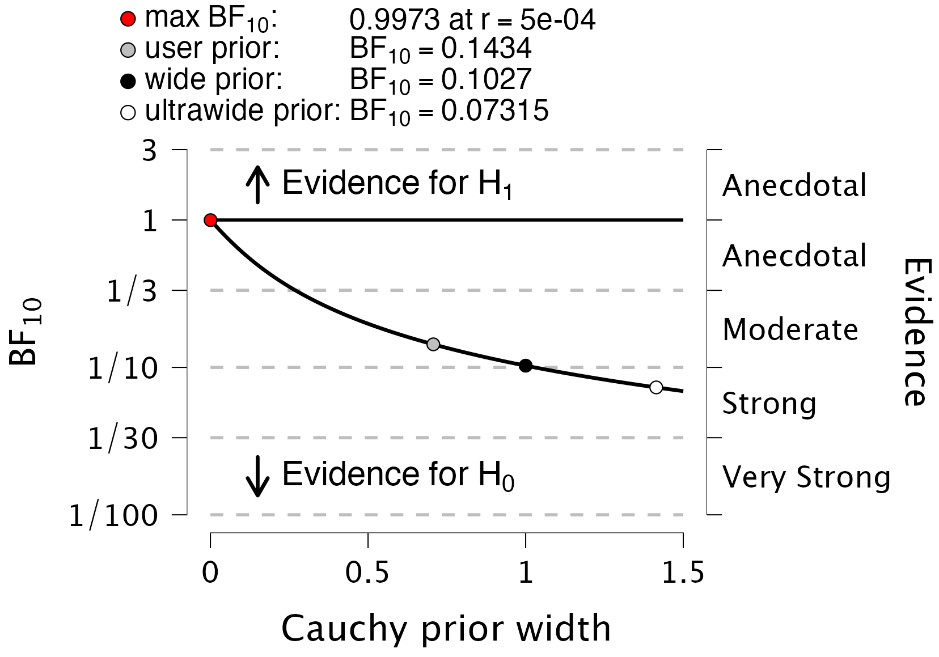


**Bayesian Independent Samples T-Test Data-driven subphenotype subgroup Diffuse malignant**

| Bayesian Independent Samples T-Test | | | | | | | | |
| --- | --- | --- | --- | --- | --- | --- | --- | --- |
| t | | n₁ | | n₂ | | BF₁₀ | |  |
| -2.000 |  | 59 |  | 59 |  | 1.171 |  |  |
|  | | | | | | | | |

**Prior and Posterior**


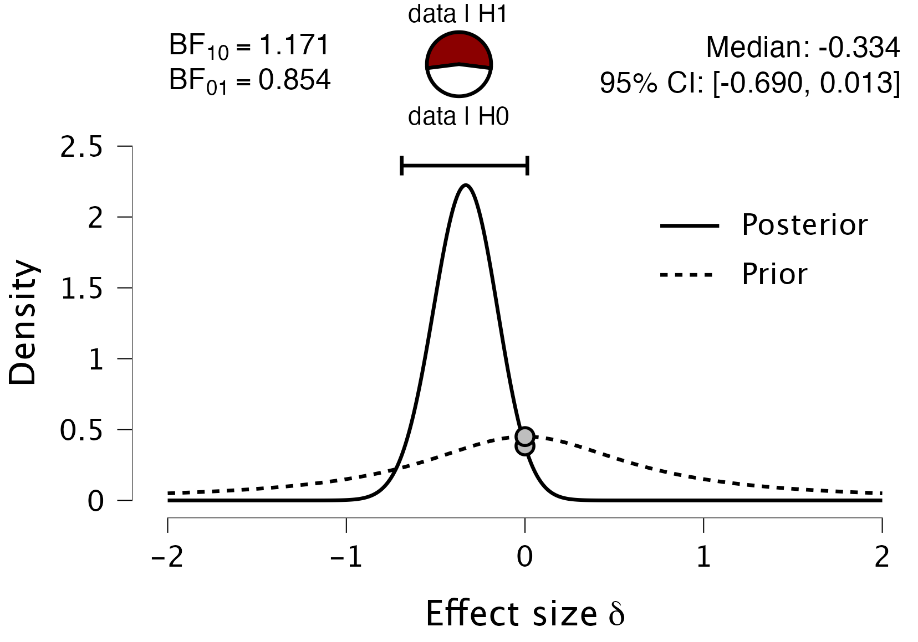


**Bayes Factor Robustness Check**


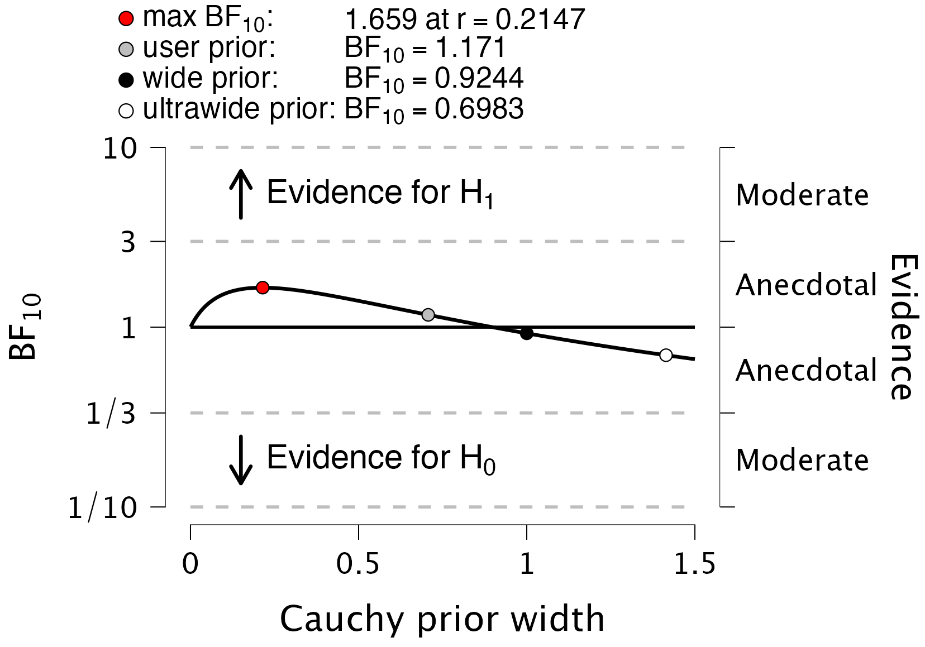


**Bayesian Independent Samples T-Test Data-driven subphenotype subgroup NonDiffuse malignant**

| Bayesian Independent Samples T-Test | | | | | | | | |
| --- | --- | --- | --- | --- | --- | --- | --- | --- |
| t | | n₁ | | n₂ | | BF₁₀ | |  |
| -0.690 |  | 257 |  | 257 |  | 0.124 |  |  |
|  | | | | | | | | |

**Prior and Posterior**


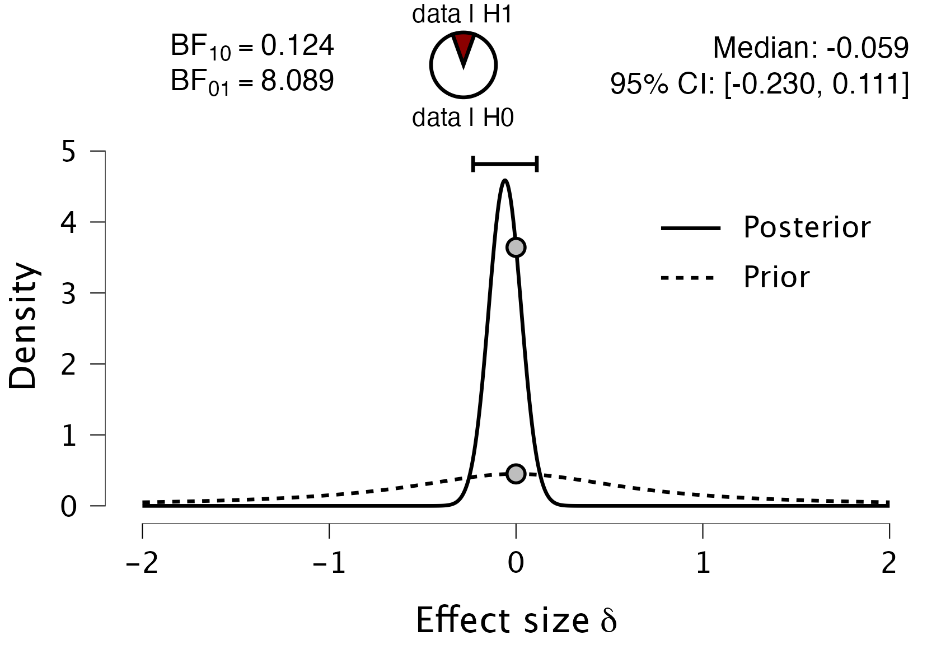


**Bayes Factor Robustness Check**


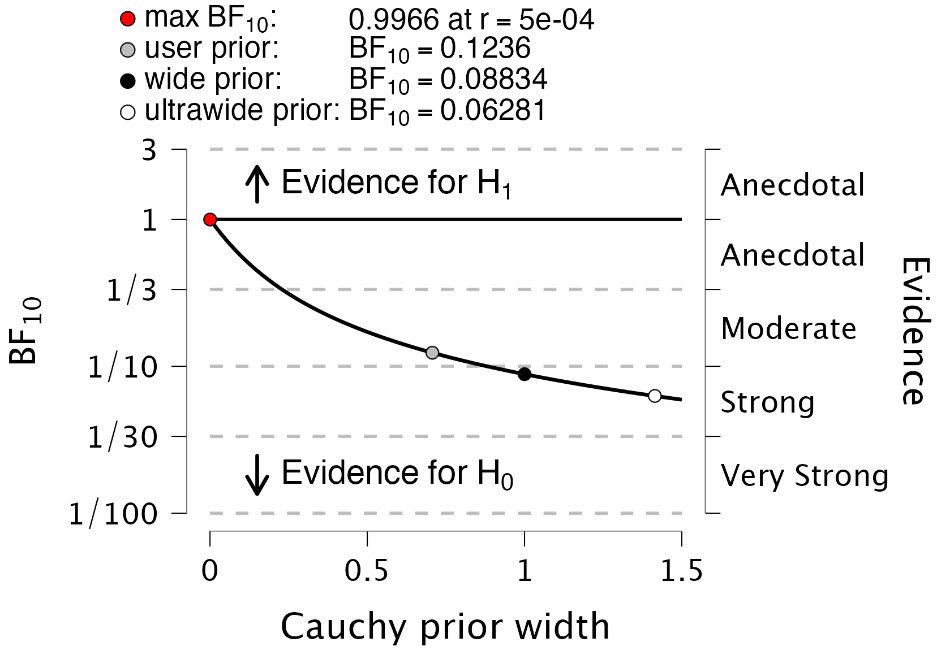

Supplement: Supplementary file 1 — Data S1. Supporting Information. [file MDS-40-619-s001.docx]
